# Predictors of permanent pacemaker implantation after sinus conversion of cavotricuspid isthmus-dependent atrial flutter

**DOI:** 10.1038/s41598-022-09439-8

**Published:** 2022-03-29

**Authors:** Juwon Kim, Sung Ho Lee, Hye Ree Kim, Tae-Wan Chung, Ji-Hoon Choi, Ju Youn Kim, Seung-Jung Park, Young Keun On, June Soo Kim, Kyoung-Min Park

**Affiliations:** 1 Division of Cardiology, Department of Medicine, Heart Vascular Stroke Institute, Samsung Medical Center, Sungkyunkwan University School of Medicine, 81 Irwon-ro, Gangnam-gu, Seoul, 06351 Republic of Korea; 2 Division of Cardiology, Department of Internal Medicine, Kangbuk Samsung Hospital, Sungkyunkwan University School of Medicine, Seoul, Republic of Korea

**Keywords:** Risk factors, Arrhythmias, Cardiology, Cardiac device therapy, Interventional cardiology

## Abstract

It is unclear which factors are associated with progressive sinus node dysfunction after cavotricuspid isthmus (CTI)-dependent atrial flutter (AFL) ablation. We sought to evaluate the incidence and predictors for permanent pacemaker (PPM) implantation after CTI-dependent AFL ablation. Between January 2011 and June 2021, 353 patients underwent CTI-dependent AFL ablation were studied. During a median follow-up of 31.6 months, 30 patients (8.5%) received PPM implantation, 24 for sick sinus syndrome and 6 for atrioventricular block. In multivariable model, prior atrial fibrillation (AF) (HR 3.570; 95% CI 1.034–12.325; P = 0.044), lowest previous sinus heart rate (HR 0.942; 95% CI 0.898–0.988; P = 0.015), and left atrial volume index (LAVI) (HR 1.067; 95% CI 1.024–1.112; P = 0.002) were independently associated with PPM implantation after CTI-dependent AFL ablation. The best cut-off points for predicting PPM implantation were 60.1 ml/m^2^ for LAVI and 46 beats per minute for lowest previous sinus heart rate. Among the patients discharged without PPM implantation after ablation, sinus pause over three seconds at AFL termination during ablation was an independent predictor of PPM implantation (HR 17.841; 95% CI 4.626–68.807; P < 0.001). Physicians should be aware of the possibility of PPM implantation during follow-up after AFL ablation, especially in patients with the relevant risk factors.

## Introduction

Previous studies have shown the clinical association between atrial tachyarrhythmia and sinus node dysfunction (SND)^1–3^. Both diseases are associated with structural and electrical atrial remodeling and stretched atria^4^. Patients with atrial fibrillation (AF) or atrial flutter (AFL) showed atrial remodeling, characterized by low voltage areas, slow conduction, and a reduction in atrial refractoriness^5–7^. Similarly, diffuse atrial and sinus node remodeling with structural changes and conduction abnormalities were also observed in patients with SND^8^. Furthermore, atrial tachyarrhythmia may directly impair sinus node function^9,10^. Importantly, previous studies demonstrated that SND might be recovered after catheter ablation for atrial tachyarrhythmia in patients with AF or AFL^5,11^. Considering reverse remodeling, the current guidelines recommended AF catheter ablation in patients with AF-related bradycardia to avoid permanent pacemaker (PPM) implantation^12^. However, even after AF catheter ablation, 8–11% of patients with AF and SND required PPM implantation during follow-up, and several studies have reported long sinus pause at the termination of AF, anterior line ablation, and high E/e’ as risk factors for progressive SND^13–15^.

The possibility of PPM implantation after AFL ablation may be commonly underestimated, unlike that of AF, since AFL is effectively treated by cavotricuspid isthmus (CTI) ablation, and CTI ablation can be performed at less equipped electrophysiology laboratories. However, some reported that patients that underwent AFL ablation were more likely to receive PPM implantations during follow-up compared with those who underwent AF ablation. Moreover, the predictors associated with irreversible or progressive SND after AFL ablation have not been reported^16,17^. Therefore, we sought to evaluate the incidence of and predictors for PPM implantation after CTI-dependent AFL catheter ablation.

## Methods

### Study population

**Figure 1:**
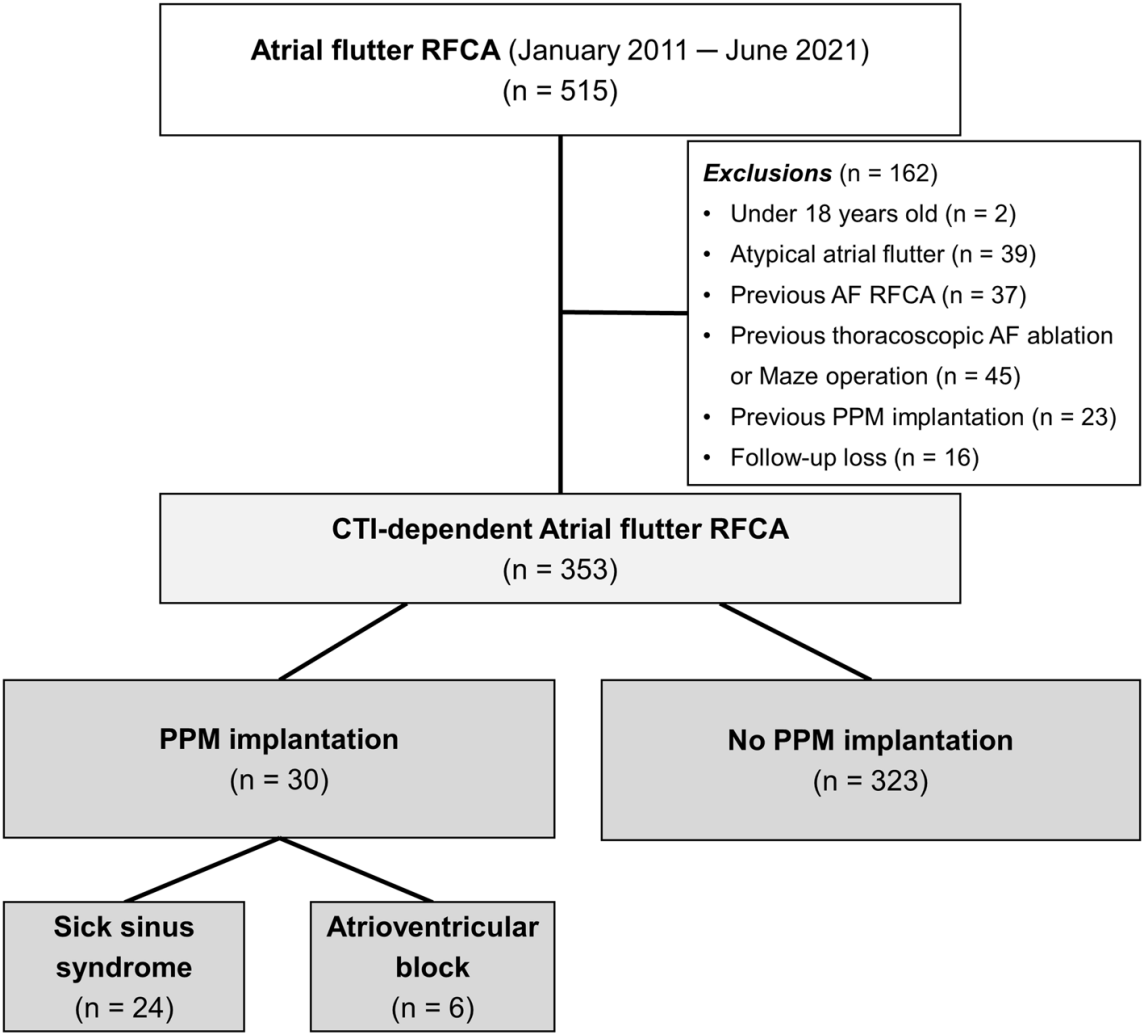
Study flow. *AF* atrial fibrillation, *CTI* cavotricuspid isthmus, *PPM* permanent pacemaker, *RFCA* radiofrequency catheter ablation.

We enrolled 515 consecutive patients who underwent AFL catheter ablation at the Samsung Medical Center (Seoul, Korea) between January 2011 and June 2021. All enrolled patients had symptomatic or drug-refractory AFL documented on electrocardiogram (ECG). Patients under the age of 18 (n = 2) and those with atypical AFL (n = 39), a previous history of AF catheter ablation (n = 37), a previous history of thoracoscopic AF ablation or Maze operation (n = 45), previous PPM implantation (n = 23), and follow-up loss (n = 16) were excluded. Finally, a total of 353 patients were selected for the current study. Among them, 30 patients underwent PPM implantation during the follow-up period (Fig. 1). The Institutional Review Board of Samsung Medical Center approved this study and waived the requirement for written informed consent. The study protocol complied with the Declaration of Helsinki.

### AFL catheter ablation procedure

All antiarrhythmic medications were withheld for at least 5 half-lives prior to the ablation procedure. The procedure was performed under conscious sedation. A duodecapolar catheter was placed into the right atrium and coronary sinus, and a His-RV catheter was placed into the His area and right ventricle apex. All catheters were inserted through the femoral vein. The surface ECG and bipolar intracardiac electrogram were simultaneously monitored and recorded. Regarding CTI ablation, an 8 Fr long sheath (RAMP or SL1, St. Jude Medical, Minneapolis, MN, USA) and a 3.5 mm tip open irrigated ablation catheter (Thermo-Cool, Bidirectional catheter, Biosense Webster, Irvine, CA, USA) were used. While radiofrequency energy was applied for 60 s with 30 watts by point, the ablation catheter was continuously pulled back along the CTI line from the side of the right ventricle to the right atrium and inferior vena cava junction. The endpoint of ablation was bidirectional electrical block confirmed by the presence of parallel and widely split (> 110 ms) double potentials along the CTI as well as activation sequence reversal when pacing from the opposite site across the CTI. If a bidirectional block was not achieved, conduction gaps were ablated, or additional line ablation was performed.

### Data collection, follow-up, and study outcomes

Baseline characteristics, 12-lead ECG, Holter ECG, echocardiography, intracardiac electrogram during the index ablation, and clinical outcome data were prospectively collected in our ablation registry by trained research coordinators using a standardized case report form and protocol. Previous 12-lead and Holter ECG findings were analyzed based on the ECGs performed within 1 year before the index ablation. AFL was considered persistent if it sustained for at least 1 week. Prior AF was defined by the presence of documented 12-lead ECG or AF > 30 s on Holter ECG. Previous echocardiographic findings were assessed based on the most recently performed echocardiography within a year prior to the index ablation. Most of the echocardiography (70.0%) included in the current study were performed one month prior to the index ablation. Left atrial (LA) volume was measured using the biplane area-length method, and the LA volume index (LAVI) was then derived by dividing LA volume by body surface area^18^. Left ventricular ejection fraction (LVEF) was assessed using the biplane Simpson’s method. Patients were routinely followed up at 1, 3, 6, and 12 months after the index procedure and biannually thereafter. Further information was collected by telephone contact or medical records if necessary. During follow-up, PPM implantation was performed when clinically indicated, based on the current guidelines^19^. The primary outcome was PPM implantation after CTI-dependent AFL catheter ablation. The secondary outcome was future PPM implantation among the patients discharged without PPM implantation after CTI-dependent AFL catheter ablation.

### Statistical analysis

Continuous variables were analyzed using the unpaired t-test or Mann–Whitney rank-sum test and were presented as means and standard deviations or medians with interquartile ranges according to their distributions, which were checked using the Kolmogorov–Smirnov test and visual inspection of Q-Q plots. All discrete and categorical variables were presented as numbers and relative frequencies (percentages) and compared using the chi-square test or Fisher’s exact test. Cox proportional hazards regression analysis was used to calculate hazard ratios (HRs) with 95% confidence intervals (CIs) to find independent predictors for PPM implantation. Multivariable Cox proportional hazard regression models were constructed using variables with a P value < 0.10 in univariable analyses. The receiver operating characteristic curve was constructed to determine the best cut-off values of LAVI and the previous lowest sinus heart rate for predicting PPM implantation after ablation. The cumulative incidence of PPM implantation was presented as a Kaplan–Meier estimate and compared using a log-rank test or Breslow test. As a sensitivity analysis, we also assessed the independent predictors for PPM implantation for sick sinus syndrome (SSS) or atrioventricular block and for PPM implantation within 1 year of CTI-dependent AFL catheter ablation using multivariable Cox regression analysis. All analyses were two-tailed, and statistical significance was defined as P < 0.05. Statistical analyses were performed using SPSS 25.0 for Windows (SPSS-PC, Chicago, IL, USA) and R version 3.6.0 (R Foundation for Statistical Computing, Vienna, Austria).

## Results

### Baseline patient and procedural characteristics

Among a total of 353 patients, 30 patients (8.5%) received PPM implantation during follow-up. The median follow-up duration was 31.6 months (interquartile range: 11.8–63.0 months). The baseline patient and procedural characteristics are presented in Table 1. Patients who underwent PPM implantation were more likely to be women and had a higher prevalence of previous stroke and AF compared to those without PPM implantation. Moreover, CHA_2_DS_2_-VASc scores were significantly higher in the patients who underwent PPM implantation. The type of AFL was not significantly different between the patients who underwent PPM and those that did not. In ECG findings prior to the index ablation, compared to patients without PPM implantation, those who received PPM implantation showed significantly lower heart rates at both sinus and AFL rhythms and higher PR intervals. The prevalence of prior sinus pauses longer than 3 seconds was higher in the patients who received PPM implantation. In echocardiographic findings before the index ablation, LAVI was significantly greater in the PPM group compared to the no PPM group. Other parameters, including LVEF, E/e’, and right ventricular systolic pressure were not significantly different depending on PPM implantation. Acute ablation success rates and HV interval were comparable between the patients who underwent PPM and those that did not. Among the cases in which AFL was terminated during CTI ablation, sinus pauses over three seconds at AFL termination were frequently observed in the PPM group. Type of antiarrhythmic agent used after ablation were similar between the two groups. During follow-up, CTI-dependent AFL recurrence rates were not significantly different between the two groups (10.0% vs. 7.4%, P = 0.883). However, patients who underwent PPM implantation showed significantly higher AF occurrence rates after AFL ablation compared to those without PPM implantation (56.7% vs. 31.6%, P = 0.010).

**Table 1:** Baseline and procedural characteristics of the study population according to PPM implantation after CTI-dependent AFL ablation.

	Total	PPM implantation ( +)	PPM implantation (-)	P value
	N = 353	N = 30 (8.5)	N = 323 (91.5)	
**Demographics**				
Age, years	61.5 ± 14.7	65.5 ± 11.7	61.1 ± 14.9	0.119
Female sex	49 (13.9)	11 (36.7)	38 (11.8)	< 0.001
Body mass index, kg/m^2^	24.4 ± 3.1	23.5 ± 2.8	24.5 ± 3.2	0.096
**Cardiovascular risk factors**				
Diabetes mellitus	89 (25.2)	7 (23.3)	82 (25.4)	0.978
Hypertension	219 (62.0)	21 (70.0)	198 (61.3)	0.458
Chronic kidney disease*	32 (9.1)	5 (16.7)	27 (8.4)	0.237
Peripheral vascular disease	13 (3.7)	0 (0.0)	13 (4.0)	0.540
Previous myocardial infarction	22 (6.2)	0 (0.0)	22 (6.8)	0.280
Heart failure	106 (30.0)	8 (26.7)	98 (30.3)	0.832
Previous stroke	23 (6.5)	6 (20.0)	17 (5.3)	0.006
**Prior AF**	140 (39.7)	18 (60.0)	122 (37.8)	0.029
**CHA**_**2**_**DS**_**2**_**-VASc score**	2.2 ± 1.5	3.0 ± 1.9	2.1 ± 1.5	0.003
**Type of AFL**				0.543
Paroxysmal	93 (26.3)	6 (20.0)	87 (26.9)	
Persistent	260 (73.7)	24 (80.0)	236 (73.1)	
**Electrocardiographic findings**				
The lowest previous sinus heart rate (beats per minute)	69.6 ± 21.3	53.1 ± 17.5	71.2 ± 21.0	< 0.001
The lowest previous AFL heart rate (beats per minute)	91.9 ± 29.6	77.9 ± 32.0	93.2 ± 29.1	0.006
Previous sinus pause over 3 s	4 (1.1)	2 (6.7)	2 (0.6)	0.036
PR interval (ms)	184.8 ± 39.0	202.0 ± 48.9	182.9 ± 37.5	0.033
QRS duration (ms)	105.8 ± 20.2	110.0 ± 24.7	105.4 ± 19.7	0.237
Right bundle branch block	32 (9.1)	3 (10.0)	29 (9.0)	1.000
Left bundle branch block	10 (2.8)	3 (10.0)	7 (2.2)	0.058
**Echocardiographic findings**				
LA volume index (ml/m^2^)	44.6 ± 14.9	57.3 ± 16.1	43.3 ± 14.1	< 0.001
LV ejection fraction (%)	56.1 ± 12.3	57.1 ± 6.8	56.0 ± 12.7	0.431
E/e’	11.4 ± 7.4	13.6 ± 6.4	11.1 ± 7.4	0.111
RV systolic pressure (mmHg)	29.4 ± 10.1	31.4 ± 12.4	29.2 ± 9.9	0.298
**Procedural findings**				
HV interval (msec)	50.3 ± 8.6	49.4 ± 7.0	50.5 ± 9.0	0.671
Acute ablation success^†^	340 (96.3)	28 (93.3)	312 (96.6)	0.689
AFL termination during ablation	275 (77.9)	24 (80.0)	251 (77.7)	0.953
More than 3 s pause at AFL termination^‡^	37 (13.4)	15 (62.5)	22 (8.7)	< 0.001
More than 5 s pause at AFL termination^‡^	16 (5.8)	11 (45.8)	5 (2.0)	< 0.001
Pause duration at AFL termination (sec)	4.7 ± 2.0	6.1 ± 2.5	4.1 ± 1.4	0.030
**Antiarrhythmic agent usage after ablation**	101 (28.6)	8 (26.7)	93 (28.8)	0.972
Class Ic	59 (18.0)	5 (16.7)	54 (16.7)	1.000
Class III	42 (12.9)	3 (10.0)	39 (12.1)	0.967
**CTI-dependent AFL recurrence**	27 (7.6)	3 (10.0)	24 (7.4)	0.883
**AF occurrence after AFL ablation**	119 (33.7)	17 (56.7)	102 (31.6)	0.010

Values are presented as the mean ± standard deviations or number (%).

*AF* atrial fibrillation, *AFL* atrial flutter, *CTI* cavotricuspid isthmus, *LA* left atrium, *LV* left ventricle, *PPM* permanent pacemaker, *RV* right ventricle.

*Chronic kidney disease was defined as serum creatinine ≥ 2.0 mg/dl.

^†^The successful endpoint of ablation was bidirectional electrical block.

^‡^The denominator was the number of patients who underwent atrial flutter termination during ablation.

### PPM implantation

The median time to PPM implantation from AFL ablation was 57 days (interquartile range: 1–854 days). Among 30 patients who received PPM implantation, 24 patients (80%) underwent PPM for SSS, and six patients (20%) underwent PPM for atrioventricular block (Table 2). 18 patients (60%) received PPM implantation within a year of ablation. The time until PPM implantation after AFL ablation is presented in Supplemental Figure. Most patients received dual-chamber PPM, but only one patient underwent leadless pacemaker implantation due to arteriovenous fistula for hemodialysis.

**Table 2:** Details of the Patients who Underwent PPM Implantation after CTI-dependent AFL Ablation.

	Total PPM implantations	PPM implantation within 1 year of ablation	PPM implantation more than 1 year after ablation
	N = 30	N = 18 (60.0)	N = 12 (40.0)
**Time to PPM implantation **(**days**)	57 (1–854)	1 (0–35)	1182 (664–2046)
**PPM indication**			
Sick sinus syndrome	24 (80.0)	13 (72.2)	11 (91.7)
2^nd^ degree AV block	2 (6.7)	2 (11.1)	0 (0.0)
Complete AV block	4 (13.3)	3 (16.7)	1 (8.3)
**Type**			
Dual chamber (transvenous)	29 (96.7)	17 (94.4)	12 (100.0)
Leadless pacemaker	1 (3.3)	1 (5.6)	0 (0.0)
**Mode**			
DDD(R)	29 (96.7)	17 (94.4)	12 (100.0)
VVI	1 (3.3)	1 (5.6)	0 (0.0)

Values are presented as the median (interquartile range), or number (%).

*AFL* atrial flutter, *AV* atrioventricular, *PPM* permanent pacemaker.

### Independent predictors for PPM implantation

**Figure 2:**
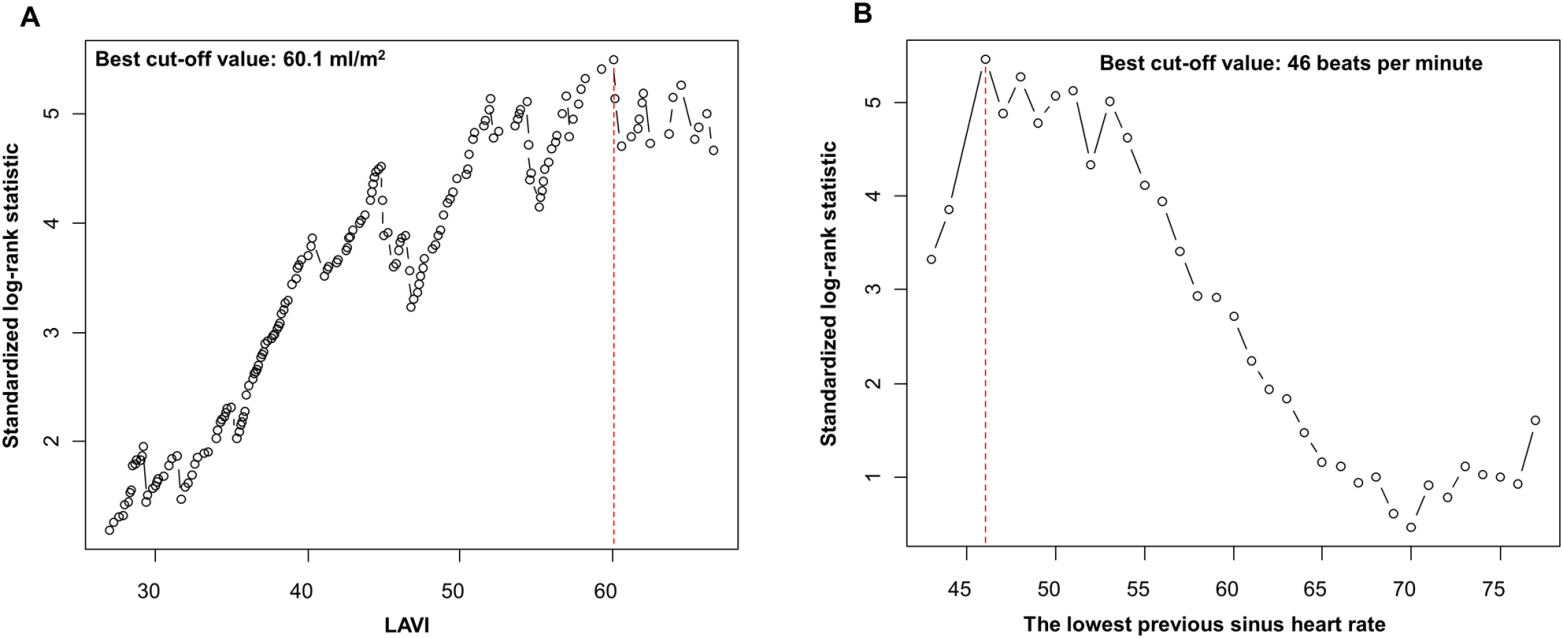
Best cut-off value of LAVI and the lowest previous sinus heart rate for the prediction of PPM implantation after CTI-dependent AFL ablation. Best cut-off value of LAVI and the lowest previous sinus heart rate to predict the risk of PPM implantation after CTI-dependent AFL ablation which was evaluated by the maximally selected log-rank statistics method. (**A**) The best cut-off value of LAVI was > 60.1 ml/m^2^ (**B**) The best cut-off value of the lowest previous sinus heart rate was < 46 beats per minute. *AFL* atrial flutter, *CTI* cavotricuspid isthmus, *LAVI* left atrial volume index, *PPM* permanent pacemaker.

**Figure 3:**
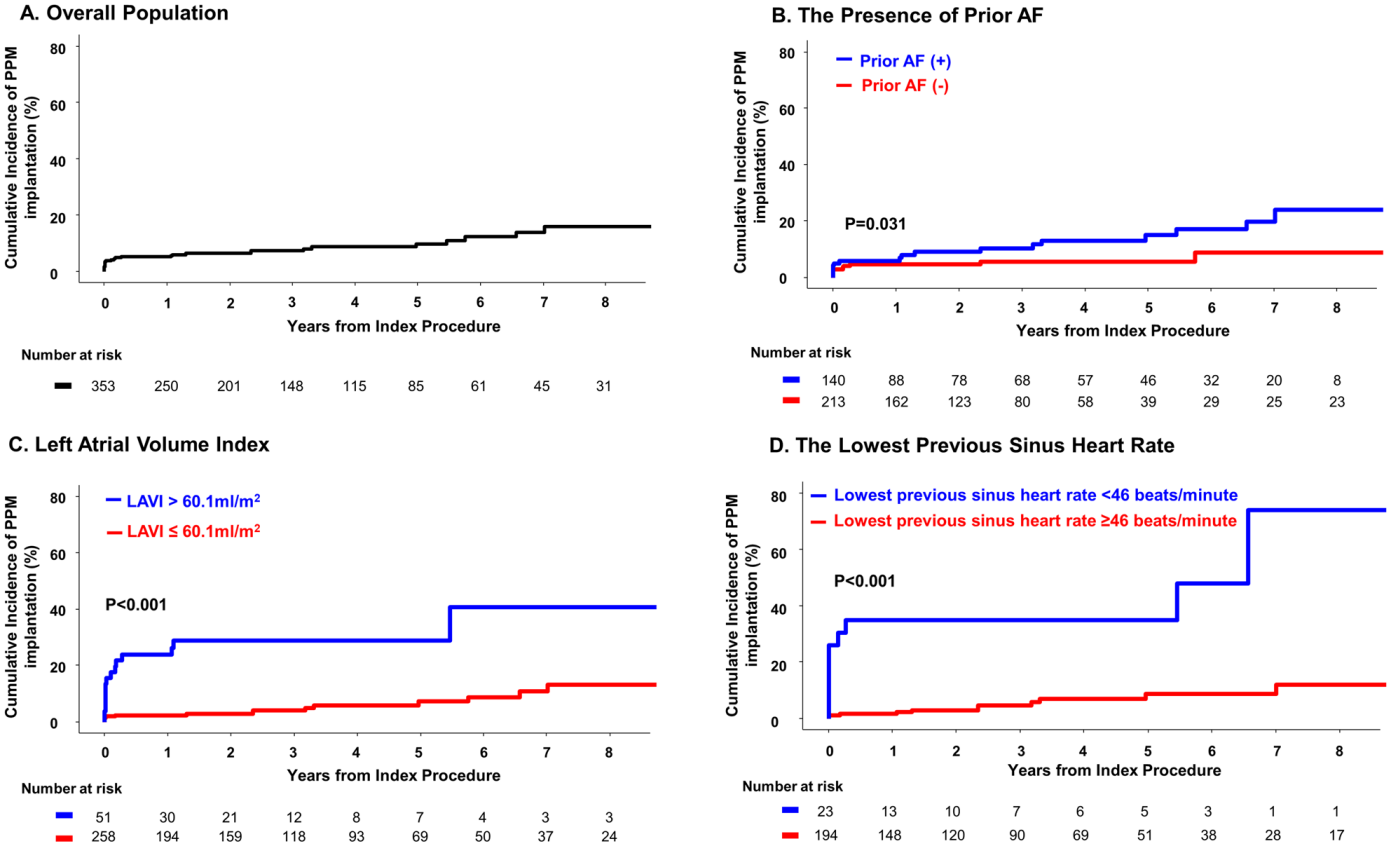
Kaplan–Meier Estimates of the Incidence of PPM Implantation after CTI-dependent AFL ablation. (**A**) Kaplan–Meier curves for PPM implantation after AFL ablation in overall population. (**B**) Kaplan–Meier curves for PPM implantation after AFL ablation according to the presence of prior AF. (**C**) Kaplan–Meier curves for PPM implantation after AFL ablation according to LAVI (cut-off value: 60.1 ml/m^2^). (**D**) Kaplan–Meier curves for PPM implantation after AFL ablation according to the lowest previous sinus heart rate (cut-off value: 46 beats per minute). *AF* atrial fibrillation, *AFL* atrial flutter, *CTI* cavotricuspid isthmus, *LAVI* left atrial volume index, *PPM* permanent pacemaker.

According to multivariable Cox regression analysis, prior AF (HR 3.570; 95% CI 1.034–12.325; P = 0.044), lowest previous sinus heart rate (HR 0.942; 95% CI 0.898–0.988; P = 0.015), and LAVI (HR 1.067; 95% CI 1.024–1.112; P = 0.002) were independently associated with PPM implantation after CTI-dependent AFL ablation (Table 3). The best cut-off points for predicting PPM implantation were 60.1 ml/m^2^ for LAVI and 46 beats per minute for the lowest previous sinus heart rate (Fig. 2). The patients with prior AF had significantly higher risk of PPM implantation after AFL ablation compared to those without prior AF (P = 0.031) (Fig. 3). The incidence of PPM implantation after AFL ablation was significantly higher in patients had LAVI > 60.1 ml/m^2^ than in patients had LAVI ≤ 60.1 ml/m^2^ (P < 0.001). The patients with the lowest previous sinus heart rate < 46 beats per minute had significantly higher risk of PPM implantation after AFL ablation compared to those with the lowest previous sinus heart rate ≥ 46 beats per minute (P < 0.001). Female sex (HR 4.310; 95% CI 1.248–14.882; P = 0.021) was an independent predictor of PPM implantation for sick sinus syndrome (Supplemental Table 1), but was not significantly associated with overall PPM implantation. Prior AF, lowest previous sinus heart rate, and LAVI were also independently associated with PPM implantation for SSS (Supplemental Table 1). However, there were no significant predictors regarding PPM implantation for atrioventricular block (Supplemental Table 2). In the sensitivity analysis, the lowest previous sinus heart rate and LAVI emerged as independent predictors of PPM implantation within a year of AFL ablation, but female sex and prior AF did not (Supplemental Table 3).

**Table 3:** Independent predictors for PPM implantation after CTI-dependent AFL ablation.

Variable	Univariate analysis		Multivariate analysis*	
	HR (95% CI)	P value	HR (95% CI)	P value
Age	1.023 (0.993–1.054)	0.133		
Female sex	4.046 (1.922–8.516)	< 0.001	2.929 (0.890–9.646)	0.077
Body mass index, per 1 kg/m^2^ increase	0.896 (0.787–1.020)	0.098	0.879 (0.694–1.113)	0.284
Diabetes mellitus	0.924 (0.397–2.154)	0.855		
Hypertension	1.430 (0.655–3.122)	0.370		
Chronic kidney disease	2.834 (1.073–7.488)	0.036	2.070 (0.308–13.927)	0.454
Heart failure	0.920 (0.408–2.073)	0.840		
Previous stroke	3.656 (1.492–8.959)	0.005	1.718 (0.223–13.229)	0.604
Prior atrial fibrillation	2.200 (1.054–4.592)	0.036	3.570 (1.034–12.325)	0.044
Persistent AFL	1.587 (0.648–3.888)	0.412		
CHA_2_DS_2_-VASc score, per 1 score increase	1.399 (1.129–1.733)	0.003	0.942 (0.599–1.481)	0.797
The lowest previous sinus heart rate, per 1 beats/minute increase	0.923 (0.891–0.957)	< 0.001	0.942 (0.898–0.988)	0.015
The lowest previous AFL heart rate, per 1 beats/minute increase	0.981 (0.968–0.995)	0.008	0.999 (0.978–1.020)	0.898
Previous sinus pause over 3 s	10.900 (2.568–46.300)	0.001	2.937 (0.326–26.468)	0.337
PR interval, per 1 ms increase	1.011 (1.001–1.022)	0.025	1.006 (0.992–1.020)	0.434
QRS duration	1.012 (0.996–1.029)	0.148		
Right bundle branch block	1.227 (0.372–4.049)	0.856		
Left bundle branch block	4.858 (1.459–16.170)	0.010	0.465 (0.051–4.261)	0.498
LA volume index, per 1 ml/m^2^ increase	1.058 (1.035–1.082)	< 0.001	1.067 (1.024–1.112)	0.002
LV ejection fraction	1.004 (0.974–1.035)	0.805		
E/e’	1.035 (1.002–1.070)	0.038	0.946 (0.857–1.043)	0.261
RV systolic pressure	1.025 (0.992–1.058)	0.140		
Type of antiarrhythmic agent used after ablation	1.011 (0.594–1.722)	0.967		

*The discriminant ability of multivariable model was 0.866 (95% CI 0.774–0.957).

*AFL* atrial flutter, *CI* confidence interval, *CTI* cavotricuspid isthmus, *HR* hazard ratio, *LA* left atrium, *LV* left ventricle, *PPM* permanent pacemaker, *RV* right ventricle.

Among the patients discharged without PPM implantation after AFL ablation, a sinus pause over three seconds at AFL termination during ablation was significantly associated with a higher risk of future PPM implantation (multivariable HR 17.841; 95% CI 4.626–68.807; P < 0.001) (Table 4).

**Table 4:** Independent Predictors for PPM Implantation among the Patients Discharged without PPM Implantation after CTI dependent AFL Ablation.

Variable	Univariate analysis		Multivariate analysis*	
	HR (95% CI)	P value	HR (95% CI)	P value
Female sex	4.044 (1.491–10.970)	0.006	1.278 (0.335–4.874)	0.720
Prior atrial fibrillation	2.617 (0.956–7.166)	0.061	1.686 (0.499–5.699)	0.400
CHA_2_DS_2_-VASc score, per 1 score increase	1.301 (0.974–1.739)	0.075	1.277 (0.833–1.957)	0.261
LA volume index, per 1 ml/m^2^ increase	1.048 (1.016–1.080)	0.003	1.039 (0.991–1.089)	0.113
More than 3 s pause at AFL termination	19.790 (5.769–67.880)	< 0.001	17.841 (4.626–68.807)	< 0.001

*The discriminant ability of multivariable model was 0.888 (95% CI 0.782–0.984).

*AFL* atrial flutter, *CI* confidence interval, *CTI* cavotricuspid isthmus, *HR* hazard ration, *LA* left atrium, *LV* left ventricle, *PPM* permanent pacemaker, *RV* right ventricle.

## Discussion

The present study evaluated the incidence of and predictors for PPM implantation after CTI-dependent AFL ablation. The major findings are as follows. First, after CTI ablation, 8.5% of patients received PPM implantation (6.8% for SSS and 1.7% for atrioventricular block) during a median follow-up period of 31.6 months. Second, prior AF, lowest previous sinus heart rate, and LAVI were independently associated with the risk of future PPM implantation after CTI ablation. Female sex was an independent predictor of PPM implantation for SSS. Third, the best cut-off values for prediction of PPM implantation were 60.1 ml/m^2^ for LAVI and 46 beats per minute for the lowest previous sinus heart rate. Fourth, among the patients discharged without PPM implantation after ablation, sinus pause over three seconds at AFL termination during ablation was an independent predictor of PPM implantation.

Atrial remodeling is considered a common mechanism for the development of atrial tachyarrhythmia and SND^4^. Importantly, atrial tachyarrhythmia (especially AF and AFL) itself may worsen sinus node function by atrial structural fibrosis, electrical remodeling, and molecular changes^5,9,10,20^. In patients with AF, atrial fibrosis with low voltage near the sinus node area was associated with SND^9^. AFL patients showed significant depression of sinus node automaticity and sinoatrial conduction^5^. In dogs that underwent atrial tachypacing, ion channel expression in sinoatrial node cells was significantly downregulated with alteration in the funny current^10^. Fortunately, atrial remodeling and SND can be reversed by ablation for AF and AFL^5,11^. In this regard, AF catheter ablation for patients with AF-related bradycardia has been incorporated into clinical practice and the current guidelines^12^. However, 8–11% of patients with AF and SND underwent PPM implantation during follow-up even after successful AF catheter ablation^13–15^. Some studies have reported long sinus pause on the termination of AF, anterior line ablation, and E/e’ to be independent predictors for PPM implantation after AF ablation in patients with AF and SND^14,15^. Contrary to AF catheter ablation, limited data are available regarding the incidence and risk factors for PPM implantation after CTI-dependent AFL ablation.

In the present study, 8.5% of patients received PPM implantation after CTI-dependent AFL ablation, and the main indication was SSS. Interestingly, this rate was comparable to the PPM implantation rate after AF catheter ablation in patients with AF and SND from previous studies (8–11%)^13–15^. Considering most patients in the present study did not have significant SND prior to AFL ablation, we assumed that patients who underwent CTI-dependent AFL ablation received more PPM implantation during follow-up compared with those who underwent AF catheter ablation. This finding is in line with a prior nationwide population-based study which showed that 8.4% of patients underwent PPM implantation during long-term follow-up after CTI-dependent AFL ablation, and PPM implantations were more frequent following AFL ablation than AF ablation^16^. However, since the follow-up period of the present study was shorter (median 31.6 months) that that of the previous nationwide study (mean 5.5 years), the incidence of PPM implantation after CTI ablation in the present study was higher than expected. Because the hospital where the present study was conducted is a tertiary referral hospital, it is likely that patients with longer duration of AFL and who had advanced atrial remodeling were included in this study. Medi et al. reported that patients with AFL showed more advanced remodeling in the posterior right atrium than patients with AF, and this finding might be associated with a higher rate of PPM implantation in patients who underwent AFL ablation than those who underwent AF ablation^7^. Complete atrioventricular block (CAVB) occurred in 1.1% (4/353) of all enrolled patients. Among them, three patients were diagnosed with CAVB shortly after CTI ablation and underwent PPM implantation during the index hospitalization. These patients showed AFL with regular slow ventricular rate (35–45 beats per minute) on 12-lead ECG just before the CTI ablation, and it was finally confirmed that the patients' rhythm was CAVB during electrophysiology study for CTI ablation. In other words, there was no case where CTI ablation directly affected atrioventricular node conduction.

The identification of risk factors for irreversible and progressive SND after AFL ablation is of great importance to the prevention of adverse events like syncope and sudden cardiac death. The present study demonstrated that prior AF, slow intrinsic sinus heart rate, and large left atrial volume significantly increased the risk of PPM implantation after AFL ablation. In this study, AF occurrence after CTI ablation was significantly higher in the patient with prior AF than without prior AF (46.4% vs. 25.4%, P < 0.001). Seara et al. also showed that prior AF was an independent predictor of transition to AF after CTI-dependent AFL ablation (HR 2.55; 95% CI 1.85–3.52, P < 0.001)^21^. Previous studies showed that enlarged LA volume was associated with a significantly increased risk of incident AF after CTI-dependent AFL ablation^22,23^. Based on these findings, the higher occurrence of AF after AFL ablation in patients with prior AF or enlarged LA volume might exacerbate SND, which results in PPM implantation during follow-up. Actually, the PPM group showed significantly higher AF occurrence rate after CTI ablation than the no PPM group. In this context, more intensive monitoring of AF occurrence after AFL ablation in patients with prior AF or enlarged LA volume may help avoid PPM implantation by providing an opportunity for early AF catheter ablation. While 39.7% of enrolled patients had documented AF before CTI ablation, CTI-dependent AFL was dominant and AF was rarely documented (less than 3 times of paroxysmal AF event) in these patients. Since CTI ablation is relatively simple procedure than AF ablation and AF ablation is generally recommended as a second-line therapy after failure of antiarrhythmic agents^12^, in the patients with typical AFL with rarely documented paroxysmal AF, we performed CTI ablation first, and then, if symptomatic AF occurred during follow-up even with antiarrhythmic agent use, we proceed AF ablation. However, considering higher AF occurrence in patients with prior AF, AF ablation at the time of CTI ablation might reduce sinus node dysfunction. Further research is warranted to confirm whether catheter ablation for AF and AFL together in the patients with typical AFL with rarely documented paroxysmal AF reduces the risk of future PPM implantation.

Additionally, the lowest previous sinus heart rate and LAVI emerged as independent predictors of relatively early PPM implantation within a year of AFL ablation in the present study. This finding suggests that patients with lower intrinsic sinus heart rate (less than 46 per minute) or greater LAVI (over 60.1 ml/m^2^) might have extensive and irreversible atrial remodeling^24^. A gender difference in the indications for PPM implantation has been shown in previous large registry studies consistently reporting female patients to have more SSS and fewer atrioventricular blocks compared to male patients^25–27^. This finding is line with the result of the present study. The mechanism of this gender disparity remains unclear, but differences in electrophysiological properties and the effects of sex hormones on ion channels might be associated with this finding^28^. Interestingly, there was a large difference in the incidence of sinus pause between before and at the time of CTI ablation. This could be explained in two ways. First, since the majority of enrolled patients had persistent AFL, sinus pause could not be detected before the CTI ablation. Thus, sinus pause was revealed after AFL termination by CTI ablation in patients with persistent AFL. Second, since the patients' rhythm was assessed intermittently with 12-lead ECG or holter at the outpatient clinic, the detection rate of sinus pause might have been low.

CTI ablation for AFL is commonly considered an effective and safe procedure. However, physicians should be aware of the possibility of PPM implantation during follow-up after AFL ablation, especially in patients with the relevant risk factors.

## Study limitations

Some limitations of the present study should be acknowledged. First, the study was a small-numbered, observational, single-center study. Potential selection bias could have influenced the study outcome. Second, the timing of echocardiography analyzed in the study was different for each patient because previous echocardiographic findings were assessed based on the most recently performed echocardiography during a year prior to the index ablation. However, most of the echocardiography (70.0%) included in the current study were performed 1 month prior to the index ablation. Third, there could be inter-operator variability in the measurement of echocardiographic parameters, including LAVI. Fourth, we did not have direct information regarding AFL or AF duration.

## Conclusion

In patients who underwent CTI-dependent AFL ablation, prior AF, slow intrinsic sinus heart rate, and large left atrial volume were independent predictors for future PPM implantation. Patients with predisposing factors for irreversible and progressive SND should be carefully monitored after AFL ablation.

## Supplementary Information

Supplementary Information.

## Data Availability

The data that support the findings of this study are available from the corresponding author upon reasonable request.
