# Supplementary material for: Predictors of permanent pacemaker implantation after sinus conversion of cavotricuspid isthmus-dependent atrial flutter
Source: Sci Rep. 2022 Mar 29;12:5336. doi: 10.1038/s41598-022-09439-8 (PMC8964688; doi:10.1038/s41598-022-09439-8)
Supplement: Supplementary file 1 — Supplementary Information. [file 41598_2022_9439_MOESM1_ESM.docx]

**Supplementary Appendix**

**Predictors of Permanent Pacemaker Implantation after Sinus Conversion of Cavotricuspid Isthmus-dependent Atrial Flutter**

**Contents**

- **Supplemental Figure.** Time until PPM implantation after CTI-dependent AFL ablation
- **Supplemental Table 1.** Independent Predictors for PPM Implantation due to Sick Sinus Syndrome after CTI-dependent AFL Ablation
- **Supplemental Table 2.** Independent Predictors for PPM implantation due to atrioventricular block after CTI-dependent AFL Ablation
- **Supplemental Table 3.** Independent Predictors for PPM Implantation within 1 Year of CTI-dependent AFL Ablation

**Supplemental Figure. Time until PPM implantation after CTI-dependent AFL ablation**


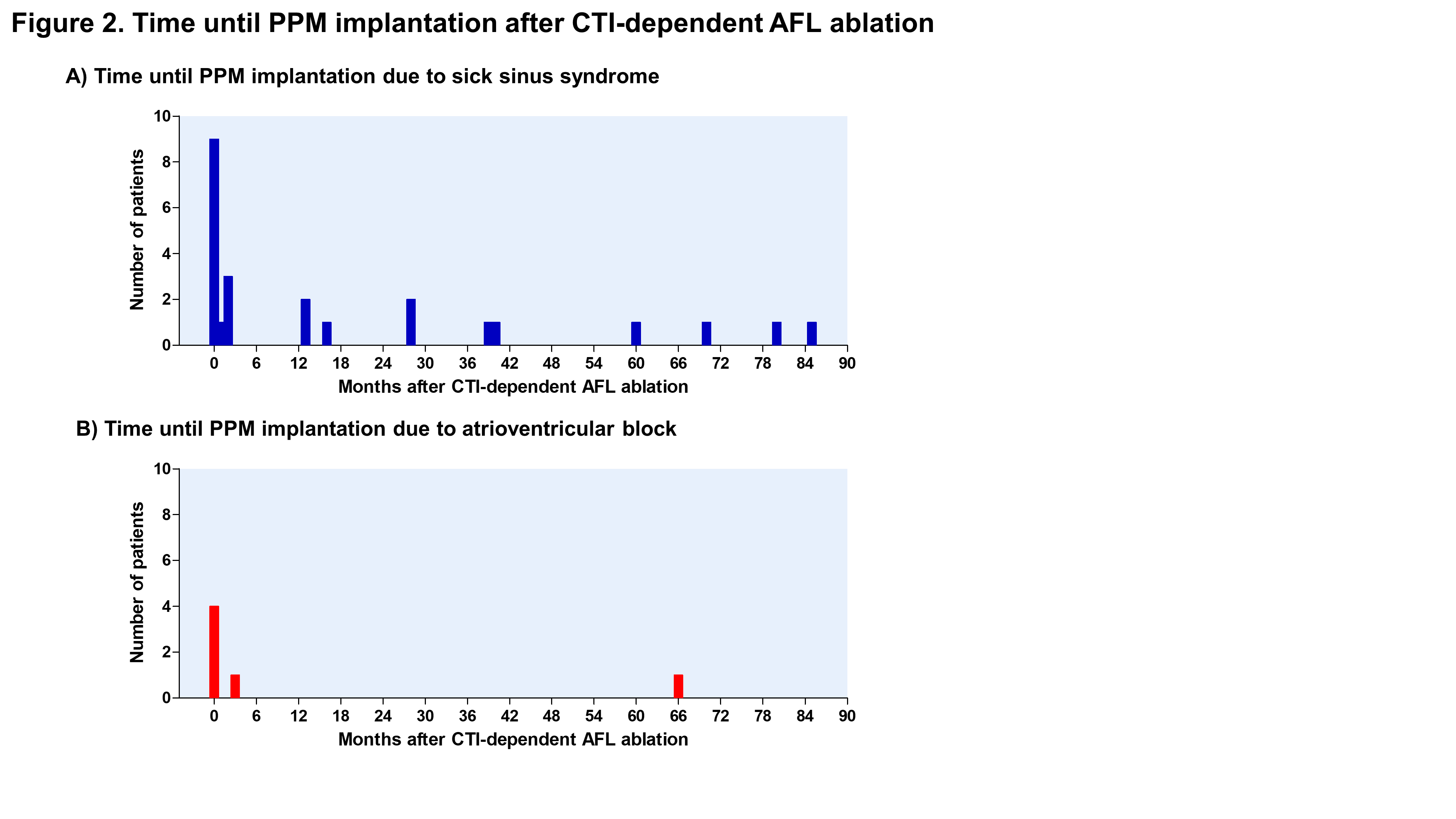


**(A)** Time until PPM implantation due to sick sinus syndrome, **(B)** Time until PPM implantation due to atrioventricular block

AFL, atrial flutter; CTI, cavotricuspid isthmus; PPM, permanent pacemaker

**Supplemental Table 1. Independent Predictors for PPM Implantation due to Sick Sinus Syndrome after CTI-dependent AFL Ablation**

| **Variable** | **Univariate analysis** | | **Multivariate analysis**^*^ | |
| --- | --- | --- | --- | --- |
|  | **HR (95% CI)** | **P value** | **HR (95% CI)** | **P value** |
| Age | 1.026 (0.991-1.061) | 0.144 |  |  |
| Female sex | 4.233 (1.848-9.694) | <0.001 | 4.310 (1.248-14.882) | 0.021 |
| Body mass index, per 1 kg/m^2^ increase | 0.843 (0.727-0.978) | 0.024 | 0.764 (0.566-1.032) | 0.080 |
| Diabetes mellitus | 0.979 (0.405-2.574) | 0.964 |  |  |
| Hypertension | 1.493 (0.619-3.601) | 0.372 |  |  |
| Chronic kidney disease | 2.956 (0.996-8.777) | 0.051 | 1.641 (0.299-9.002) | 0.568 |
| Heart failure | 0.844 (0.333-2.134) | 0.719 |  |  |
| Previous stroke | 2.082 (0.620-6.991) | 0.236 |  |  |
| Prior atrial fibrillation | 2.948 (1.255-6.929) | 0.013 | 4.268 (1.096-16.627) | 0.036 |
| Persistent AFL | 1.520 (0.567-4.077) | 0.405 |  |  |
| CHA_2_DS_2_-VASc score, per 1 score increase | 1.340 (1.053-1.705) | 0.017 | 0.664 (0.433-1.020) | 0.062 |
| The lowest previous sinus heart rate, per 1 beat/minute increase | 0.937 (0.901-0.975) | 0.001 | 0.888 (0.830-0.950) | <0.001 |
| The lowest previous AFL heart rate, per 1 beat/minute increase | 0.992 (0.978-1.006) | 0.268 |  |  |
| Previous sinus pause over 3 seconds | 14.500 (3.358-62.590) | <0.001 | 0.767 (0.110-5.372) | 0.789 |
| PR interval, per 1 ms increase | 1.003 (0.991-1.015) | 0.631 |  |  |
| QRS duration | 0.998 (0.976-1.019) | 0.829 |  |  |
| Right bundle branch block | 0.481 (0.065-3.566) | 0.474 |  |  |
| Left bundle branch block | 1.940 (0.260-14.480) | 0.518 |  |  |
| LA volume index, per 1 ml/m^2^ increase | 1.048 (1.022-1.074) | <0.001 | 1.050 (1.010-1.092) | 0.013 |
| LV ejection fration | 1.001 (0.969-1.035) | 0.931 |  |  |
| E/e’ | 1.027 (0.984-1.073) | 0.221 |  |  |
| RV systolic pressure | 1.032 (0.998-1.068) | 0.100 |  |  |
| Type of antiarrhythmic agent used after ablation | 1.043 (0.579-1.879) | 0.889 |  |  |

^*^The discriminant ability of multivariable model was 0.829 (95% CI 0.712-0.946).

AFL, atrial flutter; CI, confidence interval; CTI, cavotricuspid isthmus; HR, hazard ratio; LA, left atrium; LV, left ventricle; PPM, permanent pacemaker; RV, right ventricle.

**Supplemental Table 2. Independent Predictors for PPM implantation due to atrioventricular block after CTI-dependent AFL Ablation**

| **Variable** | **Univariate analysis** | | **Multivariate analysis** | |
| --- | --- | --- | --- | --- |
|  | **HR (95% CI)** | **P value** | **HR (95% CI)** | **P value** |
| Age | 1.014 (0.953-1.079) | 0.664 |  |  |
| Female sex | 3.327 (0.609-18.180) | 0.165 |  |  |
| Body mass index, per 1 kg/m^2^ increase | 1.092 (0.866-1.378) | 0.456 |  |  |
| Diabetes mellitus | 0.588 (0.068-5.036) | 0.628 |  |  |
| Hypertension | 1.205 (0.221-6.580) | 0.829 |  |  |
| Chronic kidney disease | 2.386 (0.276-20.620) | 0.429 |  |  |
| Heart failure | 1.257 (0.229-6.912) | 0.792 |  |  |
| Prior atrial fibrillation | 0.718 (0.130-3.977) | 0.704 |  |  |
| Persistent AFL | 1.907 (0.222-16.340) | 0.556 |  |  |
| The lowest previous sinus heart rate, per 1 beats/minute increase | 0.864 (0.785-0.951) | 0.003 | 0.675 (0.357-1.277) | 0.227 |
| The lowest previous AFL heart rate, per 1 beats/minute increase | 0.916 (0.872-0.963) | 0.001 | 0.883 (0.711-1.097) | 0.260 |
| PR interval, per 1 ms increase | 1.041 (1.017-1.066) | <0.001 | 1.175 (0.921-1.498) | 0.195 |
| QRS duration | 1.052 (1.019-1.087) | 0.002 | 1.248 (0.884-1.762) | 0.207 |
| LA volume index, per 1 ml/m^2^ increase | 1.098 (1.044-1.154) | <0.001 | 1.486 (0.867-2.547) | 0.149 |
| LV ejection fration | 1.014 (0.943-1.091) | 0.706 |  |  |
| E/e’ | 1.053 (0.998-1.106) | 0.100 |  |  |
| RV systolic pressure | 0.983 (0.903-1.083) | 0.817 |  |  |
| Type of antiarrhythmic agent used after ablation | 0.894 (0.257-3.107) | 0.859 |  |  |

AFL, atrial flutter; CI, confidence interval; CTI, cavotricuspid isthmus; HR, hazard ratio; LA, left atrium; LV, left ventricle; PPM, permanent pacemaker; RV, right ventricle.

**Supplemental Table 3. Independent Predictors for PPM Implantation within 1 Year of CTI-dependent AFL Ablation**

| **Variable** | **Univariate analysis** | | **Multivariate analysis** | |
| --- | --- | --- | --- | --- |
|  | **HR (95% CI)** | **P value** | **HR (95% CI)** | **P value** |
| Age | 1.025 (0.988-1.064) | 0.184 |  |  |
| Female sex | 4.197 (1.627-10.830) | 0.003 | 3.418 (0.687-17.005) | 0.133 |
| Body mass index, per 1 kg/m^2^ increase | 0.923 (0.787-1.083) | 0.327 |  |  |
| Diabetes mellitus | 0.838 (0.276-2.546) | 0.755 |  |  |
| Hypertension | 1.589 (0.566-4.457) | 0.379 |  |  |
| Chronic kidney disease | 4.158 (1.482 -11.670) | 0.007 |  |  |
| Heart failure | 1.152 (0.433-3.070) | 0.777 |  |  |
| Previous stroke | 6.011 (2.142-16.870) | 0.001 |  |  |
| Previous atrial fibrillation | 1.312 (0.517-3.328) | 0.568 |  |  |
| Persistent AFL | 2.928 (0.673-12.740) | 0.152 |  |  |
| CHA_2_DS_2_-VASc score, per 1 score increase | 1.536 (1.165-2.024) | 0.002 | 0.756 (0.413-1.384) | 0.365 |
| The lowest previous sinus heart rate, per 1 beat/minute increase | 0.855 (0.805-0.909) | <0.001 | 0.863 (0.786-0.947) | 0.002 |
| The lowest previous AFL heart rate, per 1 beat/minute increase | 0.973 (0.955-0.992) | 0.005 | 1.005 (0.980-1.031) | 0.695 |
| Previous sinus pause over 3 seconds | 18.700 (4.270-81.900) | <0.001 | 2.982 (0.475-18.720) | 0.244 |
| PR interval, per 1 ms increase | 1.020 (1.007-1.032) | 0.002 | 1.011 (0.984-1.040) | 0.427 |
| QRS duration | 1.023 (1.004-1.042) | 0.020 | 1.011 (0.980-1.043) | 0.505 |
| Right bundle branch block | 2.075 (0.601-7.166) | 0.249 |  |  |
| Left bundle branch block | 4.443 (1.021-19.330) | 0.047 |  |  |
| LA volume index, per 1 ml/m^2^ increase | 1.070 (1.042-1.100) | <0.001 | 1.084 (1.018-1.154) | 0.012 |
| LV ejection fration | 1.002 (0.965-1.041) | 0.898 |  |  |
| E/e’ | 1.038 (1.000-1.074) | 0.100 |  |  |
| RV systolic pressure | 1.034 (0.999-1.069) | 0.102 |  |  |
| Type of antiarrhythmic agent used after ablation | 0.842 (0.408-1.739) | 0.642 |  |  |

AFL, atrial flutter; CI, confidence interval; CTI, cavotricuspid isthmus; HR, hazard ratio; LA, left atrium; LV, left ventricle; PPM, permanent pacemaker; RV, right ventricle.
